# Revascularization vs. Conservative Medical Treatment in Patients With Chronic Kidney Disease and Coronary Artery Disease: A Meta-Analysis

**DOI:** 10.3389/fcvm.2021.818958

**Published:** 2022-02-07

**Authors:** Guang-zhi Liao, Yi-ming Li, Lin Bai, Yu-yang Ye, Yong Peng

**Affiliations:** Department of Cardiology, West China Hospital, Sichuan University, Chengdu, China

**Keywords:** meta-analysis, revascularization, conservative medical treatment, coronary artery disease, chronic kidney disease

## Abstract

**Background:**

As a strong risk factor for coronary artery disease (CAD), chronic kidney disease (CKD) indicates higher mortality in patients with CAD. However, the optimal treatment for the patients with two coexisting diseases is still not well defined.

**Methods:**

To conduct a meta-analysis, PubMed, Embase, and the Cochrane database were searched for studies comparing medical treatment (MT) and revascularization [percutaneous coronary intervention (PCI) or coronary artery bypass grafting (CABG)] in adults with CAD and CKD. Long-term all-cause mortality was evaluated, and subgroup analyses were performed.

**Results:**

A total of 13 trials met our selection criteria. Long-term (with at least a 1-year follow-up) mortality was significantly lower in the revascularization arm [relative risk (RR) = 0.66; 95% CI = 0.60–0.72] by either PCI (RR = 0.61; 95% CI = 0.55–0.68) or CABG (RR = 0.62; 95% CI = 0.46–0.84). The results were consistent in dialysis patients (RR = 0.68; 95% CI = 0.59–0.79), patients with stable CAD (RR = 0.75; 95% CI = 0.61–0.92), patients with acute coronary syndrome (RR = 0.62; 95% CI = 0.58–0.66), and geriatric patients (RR = 0.57; 95% CI = 0.54–0.61).

**Conclusion:**

In patients with CKD and CAD, revascularization is more effective in reducing mortality than MT alone. This observed benefit is consistent in patients with stable CAD and elderly patients. However, future randomized controlled trials (RCTs) are required to confirm these findings.

## Introduction

As one of the major cardiovascular diseases affecting the global human population, coronary artery disease (CAD) is the major cause of death in both developed and developing countries (1). Chronic kidney disease (CKD), an independent and strong CAD risk factor, exerts great coronary artery implications and indicates higher mortality (2). Therefore, many patients with CKD and CAD require cardiovascular optimization. However, the optimal treatment of these patients is still not well defined, and a fundamental issue is whether they will fare better with myocardial

revascularization or medical therapy. Patients with CKD were excluded from most trials, and only 10 to 40% of patients with CKD and CAD undergo revascularization in clinical practice owing to concerns about acute renal injury and major bleeding events after revascularization (3). Consequently, this population [especially regarding advanced CKD and/or end-stage kidney disease (ESKD)] is underrepresented and management is still mainly extrapolated from non-CKD cohorts (4, 5). Several investigations, mainly observational investigations, have provided varied opinions on this controversial issue, and the majority of them supported revascularization. However, according to the International Study of Comparative Health Effectiveness with Medical and Invasive Approaches-Chronic Kidney Disease (ISCHEMIA-CKD), a well-known large randomized controlled trial (RCT), there was no incremental benefit of an invasive strategy in patients with stable CAD and advanced CKD. Up to now, no agreement has been achieved on the optimal treatment in this population.

Therefore, for the purpose of providing more evidence-based ideas on the treatment of patients with CAD and CKD, eligible studies were identified and included to conduct a meta-analysis investigating the long-term effects of revascularization and medical treatment (MT) in patients with CAD and CKD or ESKD. We present the following article in accordance with the Preferred Reporting Items for Systematic Reviews and Meta-Analyses (PRISMA) reporting checklist.

## Methods

### Study Selection and Data Extraction

We searched the PubMed, Embase, and the Cochrane Library databases from inception to May 3, 2021, using search terms such as “revascularization, percutaneous coronary intervention, coronary revascularization, PCI, coronary artery bypass grafting, CABG,” “drug therapy, conservative treatment, optimal medication therapy, OMT,” and “chronic kidney disease, renal failure, renal disease, kidney disease, CKD” (Supplemental Material-words search strategy). In addition, all the references of key reviews and included articles were hand-searched for potentially missed eligible studies following a snowball procedure. Discrepancies were resolved by consensus with the addition of a third reviewer. Eligible studies met the following PICOS criteria: (1) Population: adult patients with clinical diagnoses of CKD (defined as eGFR or Ccr <60 ml/min/1.73 m^2^ or dialysis dependence) and CAD [had lesions with ≥ 50% diameter stenosis or had acute coronary syndrome (ACS)]; (2) Intervention: invasive strategies including PCI or CABG; (3) Comparative intervention: conservative medical therapy referred to patients whose initial treatment strategy did not include PCI or CABG but only drug therapy; (4) Outcome: long-term (with at least a 1-year follow-up) all-cause mortality; and (5) Study design: non-RCTs and RCTs. The exclusion criteria were as follows: (1) studies in which most patients underwent renal transplantation or were formally placed on the transplant waiting list; (2) studies not written in English; and (3) registries with overlapping populations. Two reviewers (Liao G and Li Y) screened each study by title and abstract for inclusion, reviewed the full texts of studies that qualified, and then extracted the data independently. All disagreements were resolved by discussion. The characteristics extracted from each study were the year of publication, follow-up duration, number of patients enrolled, and type of invasive therapy. The variables of patients we collected included mean or median age; sex; the proportion of the patients with complicated lesions (multivessel disease or left main artery disease), dialysis dependence, stable CAD, and diabetes. A small portion of outcome data was collected from the previous meta-analysis (3). Non-RCT and RCT quality was assessed using the Newcastle–Ottawa Scale and Cochrane Collaboration's tool, respectively.

### Data Analysis

We chose Stata MP software version 15.0 to pool relative risk (RR) with a 95% CI for the endpoint, utilizing the Mentel-Haenszel method. Between-study heterogeneity was assessed by estimating *I*^2^, and a random-effects model was used to obtain the combined RRs when the *I*^2^ statistic was over 50%. In addition, a sensitivity analysis method was applied to explain the cause of heterogeneity with a “leave-one-out” approach. Publication bias was assessed visually by inspecting funnel plots.

### Subgroup Analysis

We performed subgroup analyses of patients with dialysis dependence, stable CAD, ACS, and relatively advanced age. We also compared MT with PCI and CABG.

## Results

### Study Selection and Patient Characteristics

**Figure 1:**
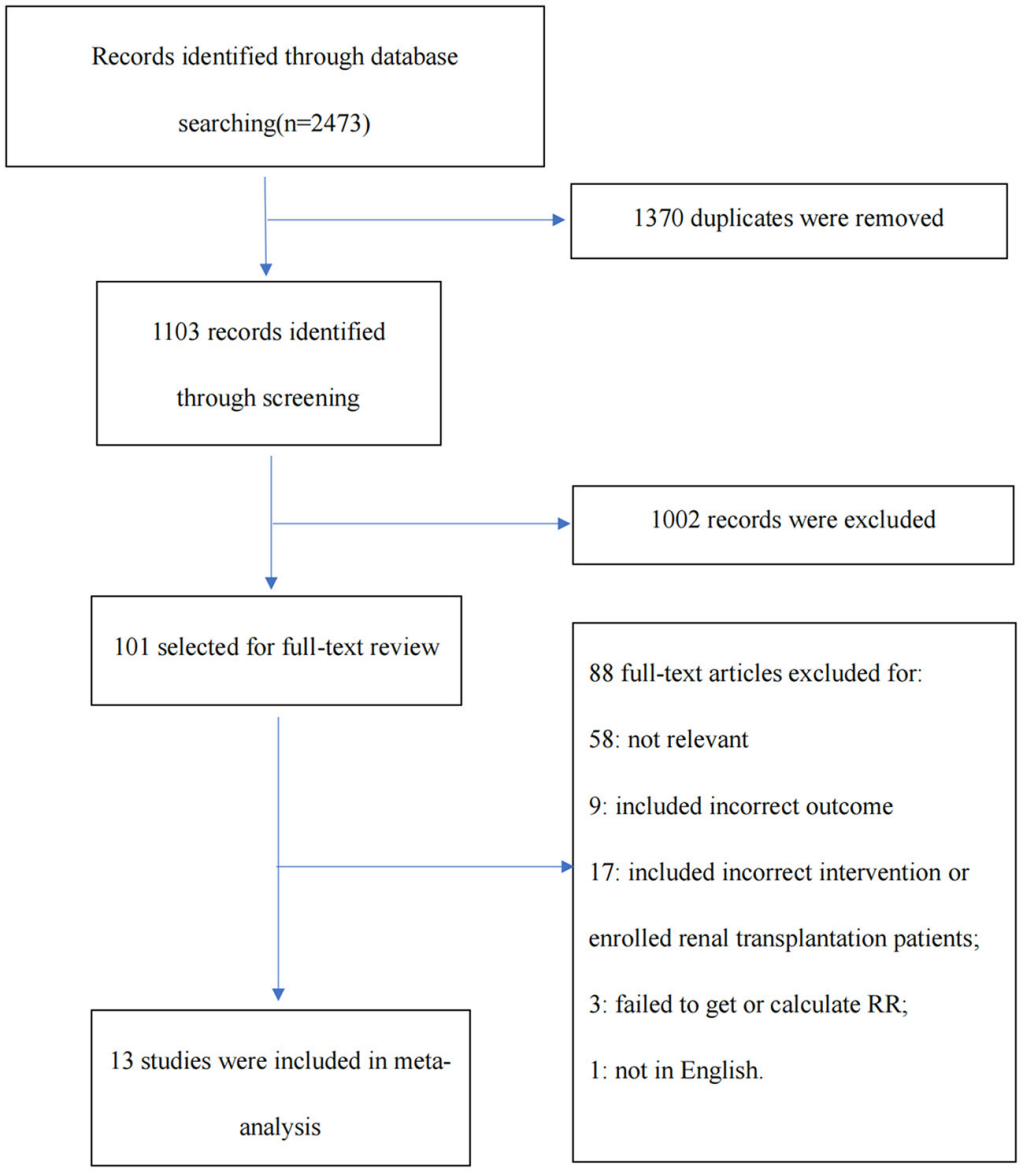
The Preferred Reporting Items for Systematic Reviews and Meta-Analyses (PRISMA) diagram illustrating the study selection process.

Our search strategy identified 2,473 records. Once duplicates had been removed, 1,103 unique records were screened, of which 101 full texts were assessed for eligibility. This process finally yielded 13 studies including two randomized controlled trials, as summarized in the PRISMA chart (Figure 1) (6–18). One trial that enrolled patients with serum creatinine (Scr) > 5 mg/dl was also included (7). The overall risk of bias was considered low in two RCTs, and the quality evaluation of non-RCTs based on the Newcastle–Ottawa scale found that all scores were ≥ 6 (Supplementary Table S2 in the supplemental material).

Study characteristics and patient demographics are summarized in Table 1. A total of 20,688 patients were included in this meta-analysis. As Table 1 shows, most of them were old (≥ 70 years old) and male patients. Diabetes is a common comorbidity, and coronary artery lesions are frequently complicated. The medications that most patients took included antiplatelet agents, β blockers, ACEIs/ARBs, and statins. In the early years, fewer patients enrolled in the trials took statins, the benefits of which have been increasingly stressed by many researchers in recent decades (19).

**Table 1:** Baseline characteristics of included studies.

**Study**	**Follow-up months**	**Types of Re**	**CKD stages**	**N**	**Age, mean or median**	**Male, %**	**Stable CAD, %**	**MVD or LAD, %**	**Dialysis, %**	**Diabetes, %**
Chertow 2000 (6)	12	PCI and CABG	Moderate	0	/	/	/	/	/	/
			Advanced	640	NA	59.0	0.0	NA	100.0	52.0
Keeley 2003 (7)	60	PCI and CABG	Moderate	1,159	65.6	52.0	0.0	NA	0.0	40.1
			Advanced	495	64.9	50.5	0.0	NA	19.0	
ICTUS 2005 (8)	12	PCI	Moderate	109	72.0	55.0	0.0	NA	0	24.1
			Advanced	8	74.9	75.0	0.0	NA	NA	37.5
Yasuda 2006 (9)	39	PCI	Moderate	0	/	/	/	/	/	/
			Advanced	134	63.3	64.2	64.9	66.4	100.0	57.4
COURAGE 2009 (10)	36	PCI	Moderate	304	68.0	77.0	100.0	NA	0.0	42.4
			Advanced	16			100.0	NA	0.0	
Eisenstein 2009 (11)	36	PCI	Moderate	1,459	74.8	48.4	NA	>70	0.0	29.3
			Advanced	0	/	/	/	/	/	/
Sakakibara 2011 (12)	44	PCI	Moderate	0	/	/	/	/	/	/
			Advanced	391	NA	NA	NA	0.0	NA	NA
Hawranek 2017 (13)	12	PCI and CABG	Moderate	5768	74.6	47.1	0.0	NA	0.0	36.7
			Advanced	1,183	76.3	40.0	0.0	NA	24.1	43.2
Kim 2018 (14)	60	PCI and CABG	NA	331	68.2	68.9	NA	>80	NA	65.0
APPROACH 2018 (15)	120	PCI and CABG	Moderate	2,157	71.8	72.8	100.0	>80	0.0	32.3
			Advanced	333	68.3	73.2	100.0	>80	35.4	56.8
Eduardo 2018 (16)	120	PCI and CABG	Moderate	150	67.0	67.3	100.0	100.0	0.0	60.0
			Advanced	0	/	/	/	/	/	/
ISCHEMIA-CKD 2020 (17)	36	PCI and CABG	Moderate	0	/	/	/	/	/	/
			Advanced	777	63.0	68.9	100.0	NA	53.4	57.1
SWEDEHEART 2009 (18)	12	PCI and CABG	Moderate	4,517	72.0	55.2	0.0	NA	0.0	31.6
			Advanced	757	71.2	57.6	0.0	NA	31.8	49.9
Total number	/	/	/	20,688	/	/	/	/	/	/

*CKD, chronic kidney disease; CAD, coronary artery disease; Re, revascularization; PCI, percutaneous coronary intervention; CABG, coronary artery bypass grafting; NA, not available; MVD, multivessel disease; LAD, left artery disease; N, number*.

### Outcome: Long-Term All-Cause Mortality

#### Revascularization vs. MT

**Figure 2:**
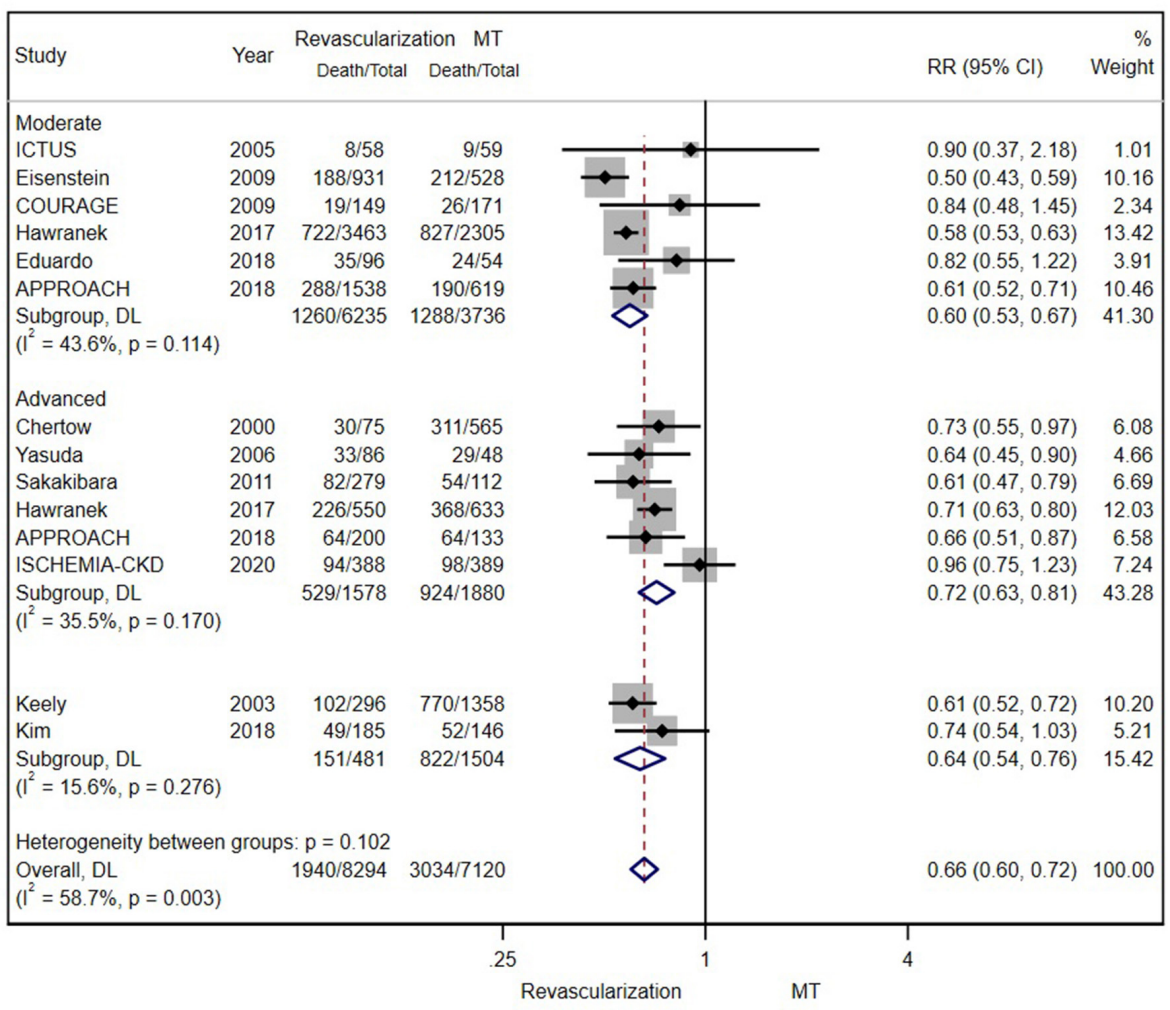
The pooled effect of revascularization and medical treatment (MT) alone on the long-term mortality of patients with coronary artery disease (CAD) and chronic kidney disease (CKD). The pooled estimate of the meta-analysis was represented with a diamond. Revascularization was more effective than MT in reducing mortality in patients with CKD. Weights and between-subgroup heterogeneity test are from random-effects model.

**Figure 3:**
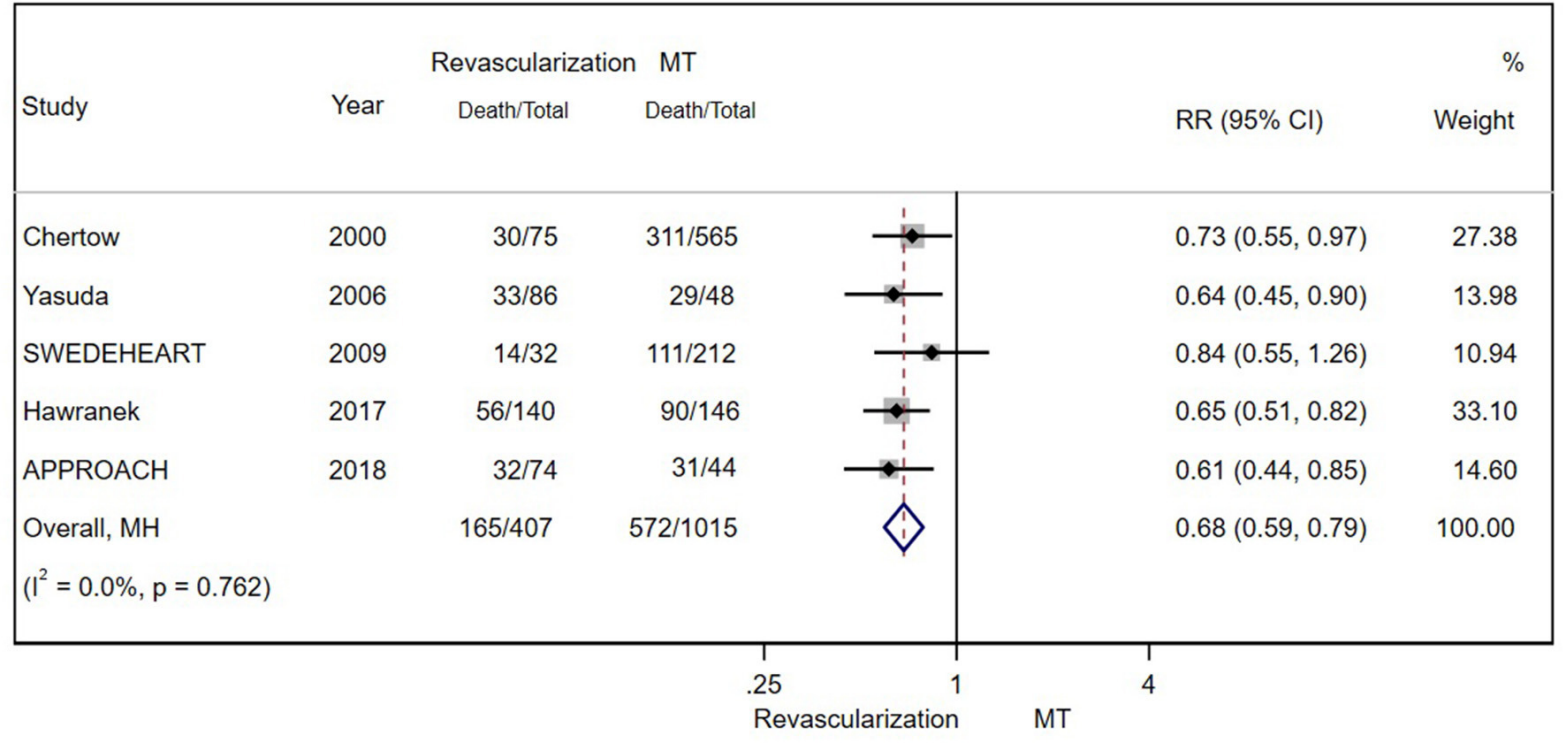
The pooled effect of revascularization and MT alone on the long-term mortality of patients with CAD and estimated glomerular filtration rate (eGFR) ≤ 15 ml/min/1.73 m^2^ or receiving dialysis treatment. Revascularization was more helpful than MT in reducing mortality. Weights and between-subgroup heterogeneity test are from Mantel-Haenszel model.

According to the original data of all-cause mortality from 12 trials (excluding SWEDEHEART 2009), we found that invasive therapy (PCI or CABG) was associated with lower long-term mortality (RR = 0.66; 95% CI = 0.60–0.72) than conservative MT (Figure 2). Subgroup analyses based on renal function categories, the moderate CKD group (RR = 0.60; 95% CI = 0.53–0.67), the advanced CKD group (RR = 0.72; 95% CI = 0.63–0.81) and even dialysis group (or with eGFR ≤ 15 ml/min/1.73 m^2^) (RR = 0.68; 95% CI = 0.59–0.79) (Figure 3), also revealed the consistent survival benefit of revascularization.

#### Subgroup Analyses

##### Percutaneous Coronary Intervention vs. MT and CABG vs. MT

**Figure 4:**
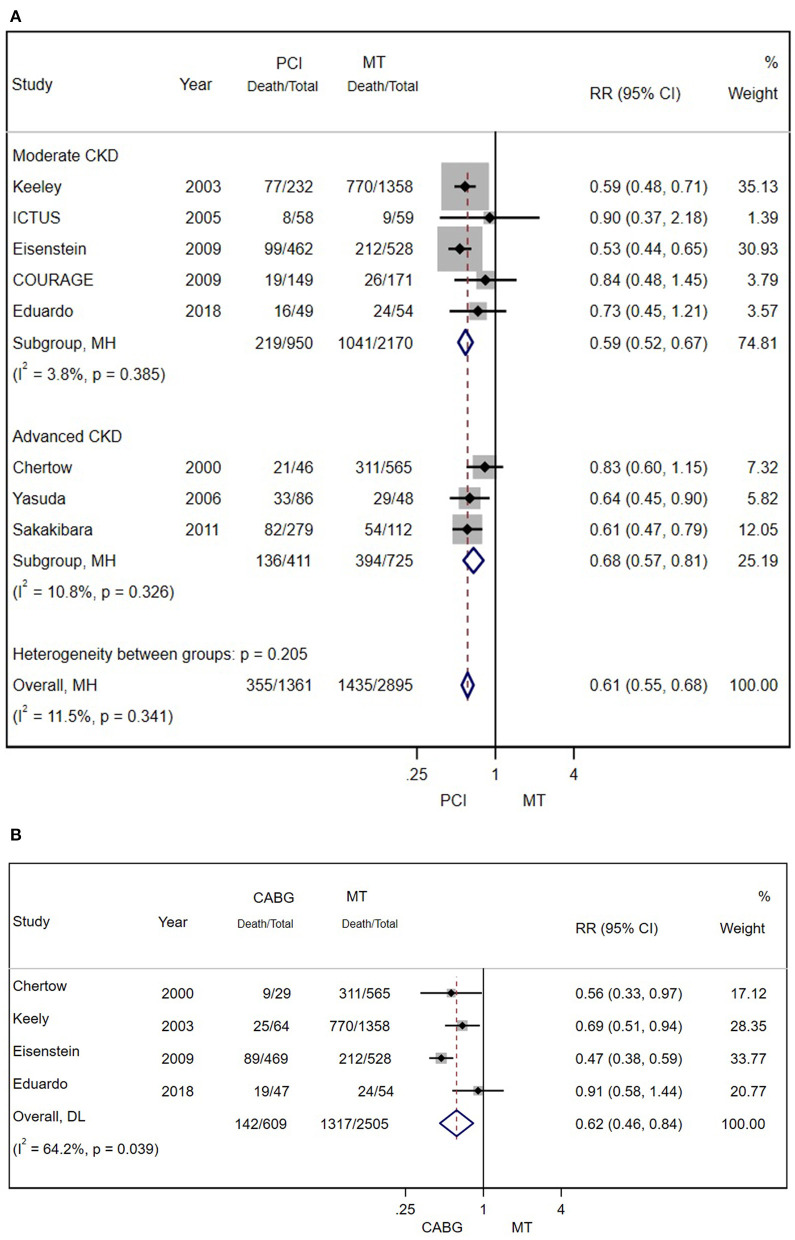
**(A)** Pooled effect of percutaneous coronary intervention (PCI) and MT alone on the long-term mortality of patients with CAD and CKD. Compared with MT alone, PCI was more effective in reducing mortality. Weights and between-subgroup heterogeneity test are from Mantel-Haenszel model. **(B)** The pooled effect of CABG and MT alone on the long-term mortality of patients with CAD and CKD. Compared with MT alone, PCI was more effective in reducing mortality. Weights and between-subgroup heterogeneity test are from random-effects model.

There were eight and four studies comparing PCI and CABG with MT in patients with CAD and CKD, respectively. The results showed that PCI (RR = 0.61; 95% CI = 0.55–0.68) (Figure 4A) and CABG (RR = 0.62; 95% CI = 0.46–0.84) (Figure 4B) were both associated with lower mortality.

##### Stable CAD

**Figure 5:**
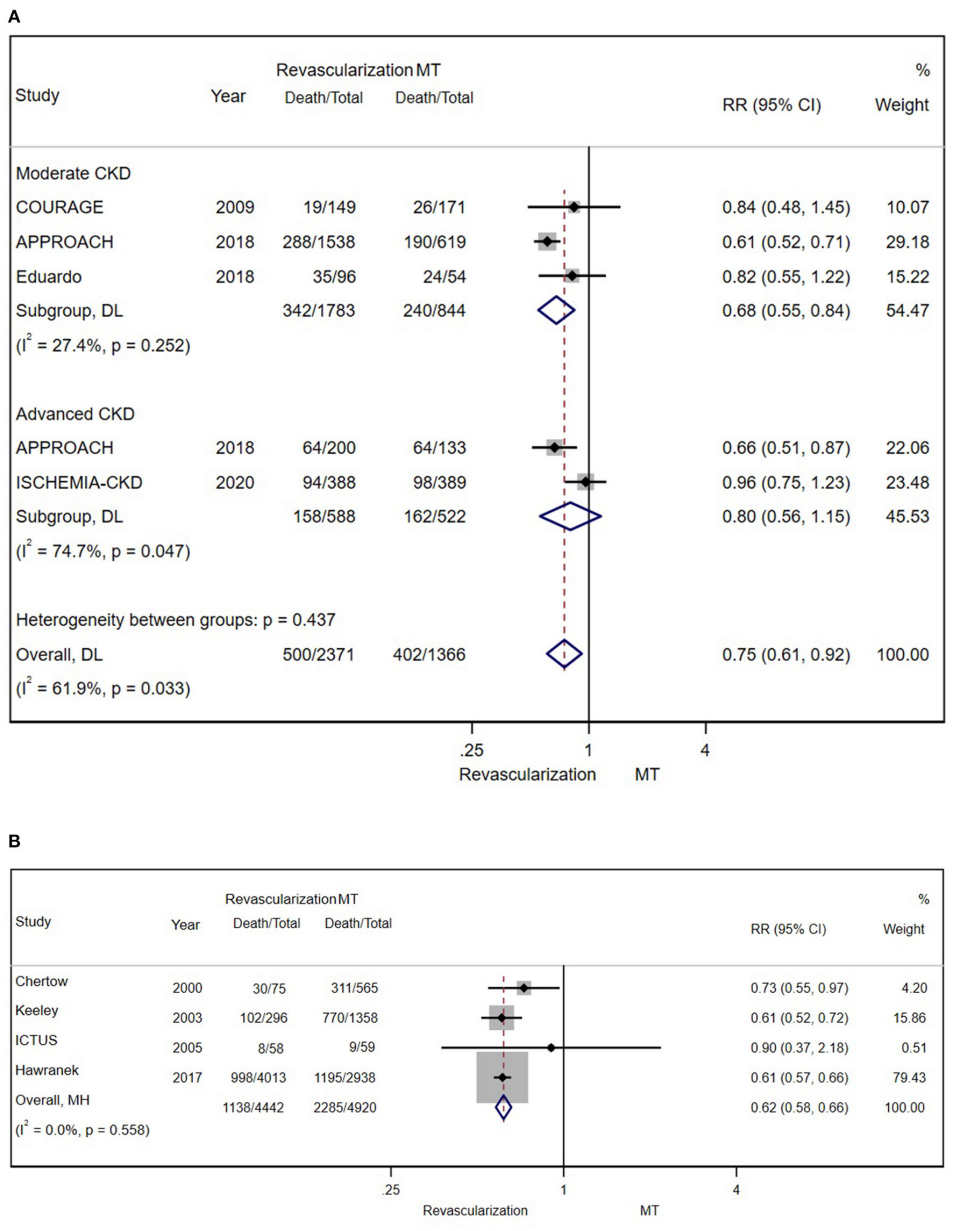
**(A)** The pooled effect of revascularization and MT alone on the long-term mortality of stable patients with CAD and CKD. Compared with MT alone, revascularization was more effective in decreasing mortality. Weights and between-subgroup heterogeneity test are from random-effects model. **(B)** The pooled effect of revascularization and MT alone on the long-term mortality of patients with ACS and CKD. Compared with MT alone, revascularization was more effective in reducing mortality. Weights and between-subgroup heterogeneity test are from Mantel-Haenszel model.

A total of 3,737 patients with stable CAD were separately examined. There was still a significant difference in the risk for all-cause mortality between moderate patients with CKD who underwent revascularization and those who received MT alone (RR = 0.75; 95% CI = 0.61–0.92) (Figure 5A).

##### Acute Coronary Syndrome

A total of 9,362 patients with ACS in 4 studies were enrolled and studied (after excluding SWEDEHEART 2009). Regardless of the severity of renal insufficiency, the results showed the long-term benefit of invasive therapy (RR = 0.62; 95% CI = 0.58–0.66) (Figure 5B). Further analysis of long-term outcomes in patients with different ACS types (unstable angina/NSTEMI and STEMI) was not conducted because of the insufficient number of related studies.

##### Elderly Patients

**Figure 6:**
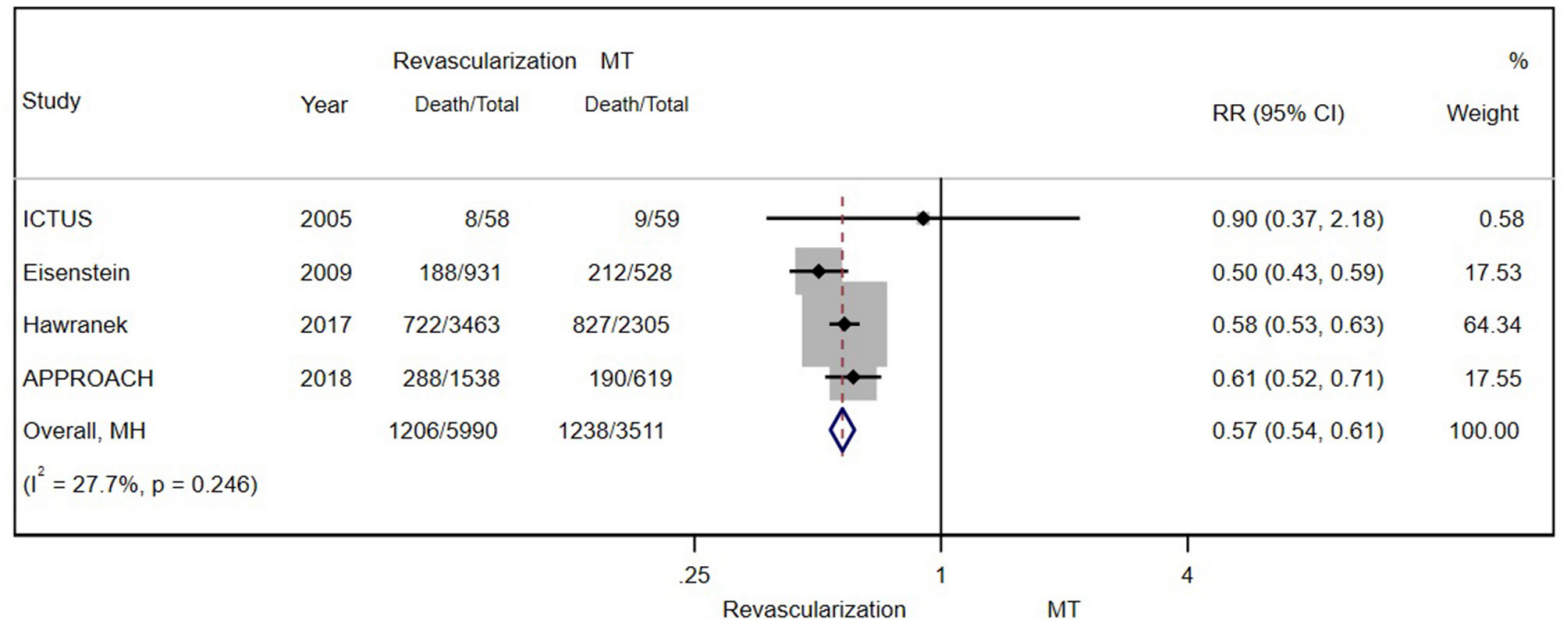
The pooled effect of revascularization and MT alone on the long-term mortality of elderly patients with CAD and moderate CKD. Compared with MT alone, revascularization was more effective in reducing mortality. Weights and between-subgroup heterogeneity test are from Mantel-Haenszel model.

The mean/median age was over 70 years in four trials (after excluding SWEDHEART 2009), so we performed a subgroup analysis of these enrolled patients. As the figure shows, revascularization was associated with a reduction in long-term mortality (RR = 0.57; 95% CI = 0.54–0.61) (Figure 6).

### Sensitivity Analysis and Publication Bias

**Figure 7:**
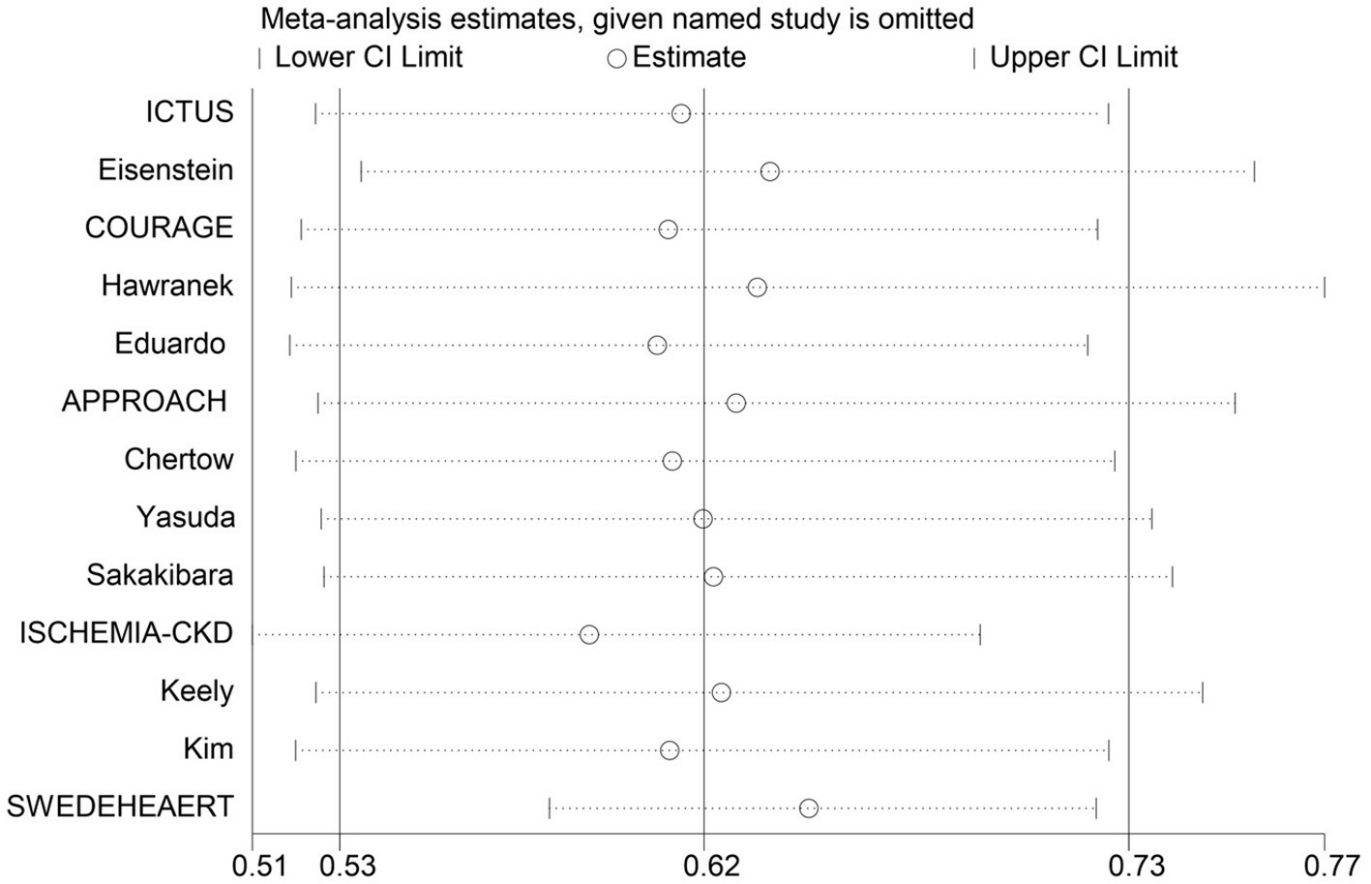
Sensitivity analysis examining the influence of individual studies on relative risk. The sequential exclusion of each study had no obvious effect on the result, indicating the robustness of our result.

Applying a “leave-one-out” approach, we found that excluding anyone did not exert a significant impact on the result (Figure 7). However, the exclusion of SWEDEHEART 2009, a study with high weight, yielded a significantly lower *I*^2^ value (Supplementary Table S1 in the supplemental material). Consistently, the SWEDEHEART 2009 study also had a similar influence on *I*^2^ in two subgroup studies of ACS and geriatric patients (Supplementary Table S1 in the supplemental material). Given, the excessively significant heterogeneity, we finally excluded the SWEDEHEART 2009 study from analyses of all eligible patients, patients with ACS, and geriatric patients.

**Figure 8:**
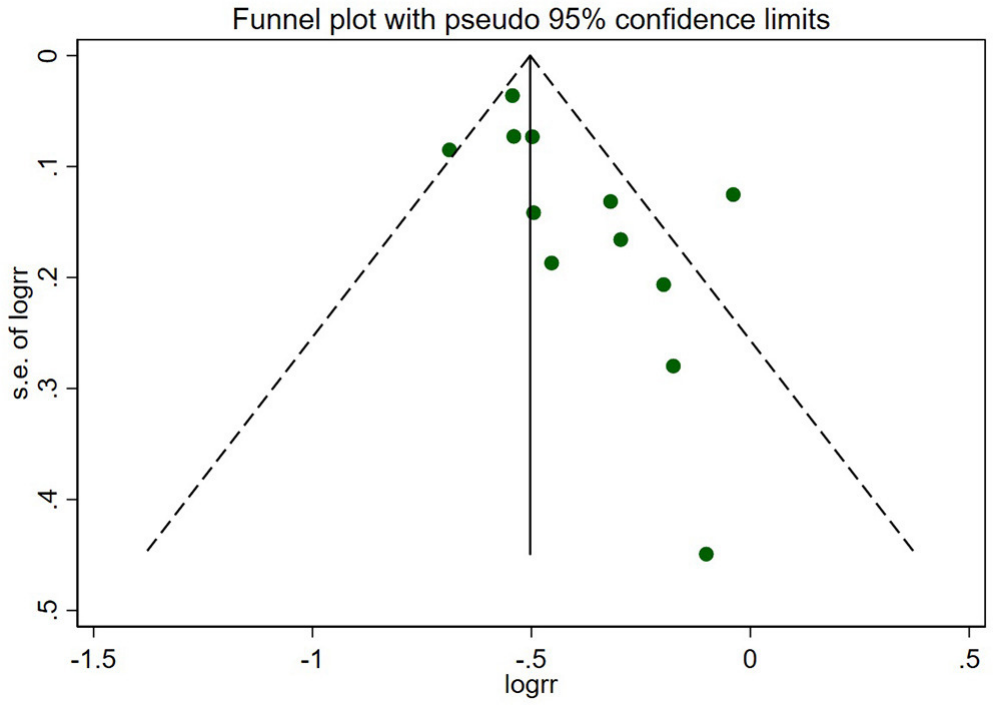
Funnel plot of the primary outcome. The circles represent the included studies. The plot suggested that there may be publication bias in this review.

As Figure 8 shows, the funnel plot suggested that there may be publication bias in our meta-analysis.

## Discussion

The principal findings of our meta-analysis are as follows: (1) Compared with drug therapy alone, revascularization (by either PCI or CABG) decreased the long-term risk for all-cause mortality in patients with CAD and CKD despite the severity of renal impairment; (2) Invasive therapy also yielded a consistent survival benefit in the subgroups with a mean age of over 70 years. Nearly, all of them were patients with moderate CKD; and (3) A lower mortality associated with revascularization was observed in the stable CAD group.

Some previous meta-analyses also compared the effects of revascularization with those of MT in patients with CAD and CKD/ESKD (3, 20). However, there were some limitations in these reviews: (1) they did not exclude patients who underwent renal transplantation or were formally placed on the waiting list, although their prognosis was different; (2) some subgroup analyses were omitted; and (3) the heterogeneity was excessively high. To reduce heterogeneity, the present meta-analysis excluded studies with most patients receiving renal transplantation or on the waiting list of renal transplantation and conducted a series of subgroup analyses. In addition, we analyzed the potential causes and mechanisms leading to our results. Consistent with the conclusions of a previous meta-analysis, our findings further supported invasive therapy. In addition, our subgroup analyses may provide new ideas for the researchers and physicians in the future.

In the revascularization era, the benefits of invasive therapy in improving patient prognosis have been accepted widely (21, 22). However, the benefits were not clear among patients with CKD (especially ESKD) because of the absence of dedicated clinical trials. Our meta-analysis suggested that positive invasive therapy regardless of PCI or CABG may predict lower long-term mortality, which can be explained by the unique characteristics of patients with CKD. On the one hand, patients with CKD were characterized by more frequently having diabetes and three-vessel CAD or left artery disease, the indications to perform revascularization (21, 23). On the other hand, a higher stage of CKD is associated with more complex coronary lesions: larger plaque burden and necrotic cores but thin less fibrous caps, which is a symbol of vulnerable plaque morphologies predisposed to plaque rupture (24–26). They all significantly increase the risk of death and other major adverse cardiovascular events. As such, these high-risk individuals with CKD may benefit from revascularization. In addition, positive intervention may also attenuate the risk of sudden death by increasing the myocyte reserve in patients with CAD to handle fluxes in fluid/electrolytes or transient changes in sympathetic tone that can otherwise result in potentially lethal arrhythmic events in patients with CKD (15, 27).

Interestingly, in contrast to the ISCHEMIA-CKD study and other studies comparing revascularization and MT in stable patients with CAD (with or without CKD) (28, 29), our results showed an association between reduced mortality and revascularization in those with CKD. However, some issues should be considered seriously in interpreting this result. In addition to the significant *I*^2^ statistic shown in Figure 5A, we noted that a negative result was yielded after the exclusion of the APPROACH study. Unlike the APPROACH study, the ISCHEMIA-CKD trial and other eligible observational studies in this subgroup analysis revealed the failure of revascularization to reduce mortality in stable patients with CAD and CKD. This difference could be explained by the considerable discrepancy in patient selection and follow-up duration. Compared with other studies, the APPROACH study enrolled a higher risk patient profile—individuals with ≥ 70% stenosis and surgical coronary disease—which was associated with the requirement of revascularization. Moreover, up to 10 years of follow-up possibly suggested late gains of revascularization. In summary, owing to the significant inconsistency in outcomes caused by various reasons, our results should be explained cautiously.

The other subgroup studies might also alter a few traditional views. It is known that geriatric patients carry a greater risk of in-hospital death and bleeding events after revascularization than younger patients (30), which leads to the underuse of revascularization in this high-risk group, let alone those with CKD. However, the survival benefit of invasive therapy was presented by our analysis in elderly individuals with CKD, which was consistent with the findings of previous studies in patients with geriatric CAD (with or without CKD) (31–33). Therefore, our results might further reveal the potential benefit of revascularization in improving the prognosis in elderly patients. However, the enrolled patients were not all aged over 70 years, which limited the representativeness of our findings in geriatric patients. The effect of invasive therapy in elderly patients with CKD should be defined according to evidence from more related trials.

For maintenance hemodialysis patients or those with eGFR ≤ 15 ml/min/1.73 m^2^, PCI and CABG are frequently not favorable options in the clinical practice, as they are usually complicated with dyslipidemia, anemia, electrolyte disorders, and arterial stiffness, increasing the risk of major bleeding and mortality during or after revascularization. Our result of the hemodialysis group was consistent with the guidelines, in which revascularization was deemed appropriate for patients with MI, including NSTEMI patients with chronic nephrosis (34, 35). However, in NSTEMI patients with ESKD, especially maintenance dialysis, whether late gains can also offset early high risks after revascularization is still not clear. As a result of scarce information, we failed to perform analysis on non-STEMI patients, indicating the great significance of performing related studies on this issue.

## Limitations

We must admit that there were a few limitations in this meta-analysis: (1) Ultimately, only 13 studies were identified to perform the meta-analysis, and they were mostly non-RCTs, in which more selection and confounding biases existed. It was difficult to control the diversities in baseline characteristics between the two arms. Since the comparisons between revascularization and MT alone compose the subgroup studies in some eligible studies, the absence of original data, especially several important baseline information such as the proportion of diabetes patients and the drug use in two arms, was a significant problem; (2) The heterogeneity of renal function in patients enrolled in different studies could not be ignored. For instance, patients in the COURAGE study were characterized by moderate impairment of renal function, while the ISCHEMIA-CKD trial only enrolled patients with advanced CKD. Therefore, it may not be appropriate to include them both to conduct the subgroup analysis of the stable CAD group, although the results of the advanced CKD/dialysis groups remained consistent; (3) Several studies calculated hazard ratios (HRs) and conducted survival analyses. In our meta-analysis, the RR was pooled to compare revascularization with MT, which consequently yielded a different result. For instance, according to the SWEDEHEART 2009 study, early revascularization improved 1-year survival in patients with NSTEMI and mild–moderate renal insufficiency (30 ml/min/1.73 m^2^ < eGFR ≤ 90 ml/min/1.73 m^2^), but the observed benefit declined with the lower renal function, and there was a trend toward harm in those with end-stage renal disease or on dialysis. The result was different when we used RR to estimate the benefit of revascularization, suggesting a strong association between invasive therapy and lower mortality even in ESKD individuals. Therefore, the instability of the results showed that we must seriously explain the finding; and (4) The funnel plot (Figure 8) showed publication bias in this review, which questioned the reliability of our results.

## Conclusion

In aggregate, the current evidence indicates that revascularization (PCI or CABG) is associated with a lower risk of long-term death than MT alone in patients with CAD and CKD. This long-term benefit was also observed in the geriatric and stable CAD groups. However, more randomized trials are urgently necessary to confirm these findings. Meanwhile, future studies should focus on renal-protective strategies to better manage these high-risk patients.

## Data Availability Statement

The original contributions presented in the study are included in the article/Supplementary Material, further inquiries can be directed to the corresponding author.

## Author Contributions

G-zL conceptualized and designed the study, collected and organized the data, conducted the analyses, drafted the initial manuscript and revised it. Y-mL conducted the analyses, reviewed the included articles, and reviewed and revised the manuscript. LB and Y-yY collected and organized the data and reviewed the included articles. YP conceptualized and designed the study, coordinated and supervised data collection, and critically reviewed and revised the manuscript. All the authors read and approved the final manuscript.

## Funding

This study was supported by the Sichuan Science and Technology Program (Grant Number 2021YFS0330, Sichuan, China), the 1·3·5 project for disciplines of excellence–Clinical Research Incubation Project, West China Hospital, Sichuan University (Grant Number: 2021HXFH061, Sichuan, China), and the Post Doctor Fellow Support Funding of Sichuan University (Grant Number: 20826041E4070).

## Conflict of Interest

The authors declare that the research was conducted in the absence of any commercial or financial relationships that could be construed as a potential conflict of interest.

## Publisher's Note

All claims expressed in this article are solely those of the authors and do not necessarily represent those of their affiliated organizations, or those of the publisher, the editors and the reviewers. Any product that may be evaluated in this article, or claim that may be made by its manufacturer, is not guaranteed or endorsed by the publisher.
