# Supplementary material for: Revascularization vs. Conservative Medical Treatment in Patients With Chronic Kidney Disease and Coronary Artery Disease: A Meta-Analysis
Source: Front Cardiovasc Med. 2022 Feb 7;8:818958. doi: 10.3389/fcvm.2021.818958 (PMC8858980; doi:10.3389/fcvm.2021.818958)
Supplement: Supplementary file 1 [file Data_Sheet_1.DOCX]

**Supplemental Material**

The Comparison of Long-term Mortality between Revascularization and Medical Treatment in Patients with Coronary Artery Disease and Chronic Kidney Disease: A Meta-Analysis

Guang-zhi Liao, Yi-ming Li, Lin Bai, Yu-yang Ye, Yong Peng^*^

**Words Search Strategy**

**Table S1. The influences of SWEDEHEART 2009 on pooled effects and heterogeneity.**

**Table S2. Quality assessment**

**Words Search Strategy**

**Pubmed**

#1 Coronary Artery Disease[MeSH Terms]

#2 ((((((((Arterioscleros*[Title/Abstract]) OR (Atheroscleros*[Title/Abstract])) OR (Coronary[Title/Abstract])) OR (Ischemia[Title/Abstract])) OR (Occlusion *[Title/Abstract])) OR (STEMI[Title/Abstract])) OR (NSTEMI*[Title/Abstract])) OR (Angina[Title/Abstract])) OR (ACS[Title/Abstract])

#3 #1 OR #2

#4 Kidney Failure, Chronic[MeSH Terms]

#5 ((((Kidney[Title/Abstract]) OR (Renal[Title/Abstract])) OR (Dialysis[Title/Abstract])) OR (CKD[Title/Abstract])) OR (ESRD[Title/Abstract])

#6 #4 OR #5

#7 #3 AND #6

#8 Percutaneous Coronary Intervention[MeSH Terms]

#9 ((Intervention[Title/Abstract]) OR (PCI[Title/Abstract])) OR (Stent[Title/Abstract])

#10 Coronary Artery Bypass[MeSH Terms]

#11 ((Graft[Title/Abstract]) OR (CABG[Title/Abstract])) OR (Surgery[Title/Abstract])

#12 #8 OR #9 OR #10 OR #11

#13 Drug Therapy[MeSH Terms]

#14 (((((Medici*[Title/Abstract]) OR (Drug[Title/Abstract])) OR (Conservative[Title/Abstract])) OR (OMT[Title/Abstract])) OR (MT[Title/Abstract])) OR (Pharmacotherap* [Title/Abstract])

#15 #13 OR #14

#16 #7 AND #12 AND #15

**Cochrane Library**

#1 Coronary Artery Disease

#2 (Arteriosclerosis):ab,ti,kw OR (Atherosclerosis):ab,ti,kw OR (Coronary):ab,ti,kw OR (Ischemia):ab,ti,kw OR (Angina):ab,ti,kw OR (Ischemic):ab,ti,kw OR (Occlusion):ab,ti,kw OR (STEMI):ab,ti,kw OR (NSTEMI):ab,ti,kw OR (ACS):ab,ti,kw

#3 #1 OR #2

#4 Kidney Failure, Chronic

#5 (Kidney):ti,ab,kw OR (Renal):ti,ab,kw OR (Dialysis):ti,ab,kw OR (CKD):ti,ab,kw AND (ESRD):ti,ab,kw

#6 #4 OR #5

#7 #3 AND #6

#8 Percutaneous Coronary Intervention

#9 (Intervention):ti,ab,kw OR (PCI):ti,ab,kw OR (Stent):ti,ab,kw

#10 Coronary Artery Bypass

#11 (Graft):ti,ab,kw OR (CABG):ti,ab,kw OR (Surgery):ti,ab,kw

#12 #8 OR #9 OR #10 OR #11

#13 Drug Therapy

#14 (Drug):ti,ab,kw OR (Pharmacotherapy):ti,ab,kw OR (Medicince):ti,ab,kw OR (Medical):ti,ab,kw OR (Medication):ti,ab,kw OR (Conservative):ti,ab,kw OR (OMT):ti,ab,kw OR (MT):ti,ab,kw

#15 #13 OR #14

#16 #7 AND #12 AND #15

**Embase**

#1 Coronary Artery Disease

#2 'Arteriosclerosis':ab,ti OR 'Atherosclerosis':ab,ti OR 'Occlusion':ab,ti OR 'Ischemia':ab,ti OR 'angina':ab,ti OR ' Occlusion':ab,ti OR 'STEMI':ab,ti OR 'ACS':ab,ti

#3 #1 OR #2

#4 Kidney Failure, Chronic

#5 'Kidney':ab,ti OR 'Renal':ab,ti OR 'Dialysis:ab,ti OR 'CKD':ab,ti OR 'ESRD':ab,ti

#6 #4 OR #5

#7 #3 AND #6

#8 Percutaneous Coronary Intervention

#9 'Intervention':ab,ti OR 'PCI':ab,ti OR 'Stent':ab,ti

#10 Coronary Artery Bypass

#11 'Graft':ab,ti OR 'CABG':ab,ti OR 'Surgery':ab,ti

#12 #8 OR #9 OR #10 OR #11

#13 Drug Therapy

#14 'Drug':ab,ti OR 'Pharmacotherapy':ab,ti OR 'Medicince' OR 'Medical':ab,ti OR 'Medication':ab,ti OR 'Conservative':ab,ti OR 'OMT':ab,ti OR 'MT':ab,ti

#15 #13 OR #14

#16 #7 AND #12 AND #15

Table S1. The influences of SWEDEHEART 2009 on pooled effects and heterogeneity.

| Long-term mortality | In all the enrolled patients | In ACS group | In geriatric group |
| --- | --- | --- | --- |
| With SWEDEHEART 2009 | RR=0.63;95%CI=0.56-0.72; I^2^=81.6％ | RR=0.56; 95%CI=0.41-0.77; I^2^=93.2％ | RR=0.51; 95%CI=0.39-0.66;  I^2^=91.8％ |
| Excluding SWEDEHEART 2009 | RR=0.66; 95%CI=0.60-0.72  I^2^=58.7％ | RR=0.62; 95%CI=0.58-0.66; I^2^=0.0％ | RR=0.57; 95%CI=0.54-0.61;  I^2^=27.7％ |
| ACS: acute myocardial infarction; CI: confidence interval; RR: risk ratio | | | |

Table S2. Quality assessment

Table S2.1 Newcastle-Ottawa scale for assessing the observational studies

| Studies | Study design | Selection | Comparability | Outcome |
| --- | --- | --- | --- | --- |
| Chertow 2000 ^8^ | Prospective cohort | ★★★★ | ★★ | ★★ |
| Keeley 2003 ^12^ | Prospective cohort | ★★★★ | ★★ | ★★ |
| Yasuda 2006 ^17^ | Prospective cohort | ★★★★ | ★★ | ★★★ |
| SWEDEHEART 2009 ^16^ | Prospective cohort | ★★★★ | ★★ | ★★ |
| COURAGE 2009 ^6^ | Post-hoc analysis | ★★★ | ★★ | ★★ |
| Eisenstein 2009 ^9^ | Prospective cohort | ★★★★ | ★★ | ★★ |
| Sakakibara 2011 ^14^ | Prospective cohort | ★★★ | ★★ | ★ |
| Hawranek 2017 ^10^ | Retrospective cohort | ★★★ | ★★ | ★★ |
| Kim 2018 ^11^ | Prospective cohort | ★★★★ | ★★ | ★★ |
| APPROACH 2018 ^15^ | Prospective cohort | ★★★★ | ★★ | ★★ |
| Eduardo 2018 ^13^ | Post-hoc analysis | ★★★ | ★★ | ★★★ |

All the observational studies are assessed through a star rating system based on the selection of study subjects (maximum four stars), comparability of study groups (maximum two stars) and assessment of the outcome (maximum three stars). Studies rated with six or more stars were considered to be of high quality.

Table S2.2 Cochrane collaboration’s tool for assessing randomized controlled trials

| Study | Selection bias | Performance bias | Detection bias | Attrition bias | Reporting bias | Other bias |
| --- | --- | --- | --- | --- | --- | --- |
| Ischemia-CKD 2020^18^ | Low risk | Unclear risk | Low risk | Low risk | Unclear risk | Unclear risk |
| ICTUS 2005^7^ | Low risk | Unclear risk | Low risk | Low risk | Unclear risk | Unclear risk |
